# Prodrugs of Fluoro-Substituted Benzoates of EGC as Tumor Cellular Proteasome Inhibitors and Apoptosis Inducers

**DOI:** 10.3390/ijms9060951

**Published:** 2008-06-02

**Authors:** Zhiyong Yu, Xu Long Qin, Yan Yan Gu, Di Chen, Qiuzhi Cindy Cui, Tao Jiang, Sheng Biao Wan, Q. Ping Dou

**Affiliations:** 1 The Prevention Program, Barbara Ann Karmanos Cancer Institute, and Department of Pathology, School of Medicine, Wayne State University, Detroit, Michigan, USA; 2 Key Laboratory of Marine Drug, Ministry of Education, Medical College, Ocean University of China, Qingdao, China; 3 Shandong Tumor Hospital & Institute, Breast Cancer Center, Jinan, Shandong, China

**Keywords:** tea polyphenols, prodrugs, proteasome inhibitors, cancer prevention, cancer therapy

## Abstract

The most potent catechin in green tea is (-)-epigallocatechin-3-gallate [(-)-EGCG], which, however, is unstable under physiological conditions. To discover more stable and more potent polyphenol proteasome inhibitors, we synthesized several novel fluoro-substituted (-)-EGCG analogs, named F-EGCG analogs, as well as their prodrug forms with all of -OH groups protected by acetate. We report that the prodrug form of one F-EGCG analog exhibited greater potency than the previously reported peracetate of (-)-EGCG to inhibit proteasomal activity, suppress cell proliferation, and induce apoptosis in human leukemia Jurkat T cells, demonstrating the potential of these compounds to be developed into novel anti-cancer and cancer-preventive agents.

## 1. Introduction

Proteasome inhibitors have been considered as potential anticancer drugs [1]. Inhibition of proteasome prevents ubiquitin-targeted proteolysis which can affect multiple signaling cascades within the cell. Since disruption of normal homeostatic mechanisms can lead to cell death, the discovery of novel proteasome inhibitors with little or no toxicity is highly desirable in anticancer therapy [2–3]. The 20S proteasome, the proteolytic core of 26S proteasome complex, contains multiple peptidase activities (including the chymotrypsin-like, trypsin-like and peptidylglutamyl peptide hydrolyzing-like/PGPH) [1–3]. It has been shown that inhibition of chymotrypsin-like but not trypsin-like proteasomal activity is a strong stimulus that induces apoptosis [1–3]. We have illustrated that inhibition of proteasome may be a key mechanism for the cancer-preventive activity of green tea [4–5].

Tea leaves contain many constituents [6]. Among these constituents are the polyphenolic catechins, which are thought to contribute to the biological effect of tea [7]. The main polyphenols found in green tea extracts are (-)-epicatechin [(-)-EC], (-)-epigallocatechin [(-)-EGC], (-)-epicatechin-3-gallate [(-)-ECG], and (-)-epigallocatechin-3-gallate [(-)-EGCG]. In particular, (-)-EGCG, the most abundant catechin, was found to be the strongest chemopreventive and anticancer agent among the green tea catechins [8]. However, (-)-EGCG has at least one limitation: it has poor bioavailability, attributing to its low absorption and poor stability in neutral or alkaline solutions [9].

We have synthesized and evaluated a serial of tea polyphenol analogs, and reported that synthetically derived (-)-EGCG analogs potently inhibit the proteasomal chymotrypsin-like activity, leading to growth arrest and apoptosis [10]. We have also examined the structure–activity relationship of a number of synthetic green tea polyphenol analogs involving modifications of A ring and B ring of (-)-EGCG as proteasome inhibitors. It was found that in B ring, a decrease in the number of OH groups led to decreased potency [11].

Most recently, in order to discover more stable and more potent tea polyphenols as proteasome inhibitors, we synthesized several novel fluoro-substituted benzoates of EGC, or (-)-EGCG analogs with eliminated −OH groups from the D-ring and replaced with one or two fluorine(s), named F-EGCG analogs (Sun Dong Kui et al, to be submitted). In addition, we synthesized peracetates of these F-EGCG analogs since we reported that the peracetate-protected or prodrug form of (-)-EGCG, Pro-EGCG (1), was more stable and more potent than natural (-)-EGCG *in vitro* and *in vivo* [12]. We found that, compared to Pro-EGCG (1), the peracetate of 3,4-difluorobenzoates of EGC (Pro-F-EGCG4) exhibited greater potency to inhibit proteasomal chymotrypsin-like activity, suppress cell proliferation and induce apoptosis in human leukemia Jurkat T cells.

## 2. Results and Discussion

### 2.1. Inhibition of the chymotrypsin-like activity of a purified 20S proteasome by synthetic F-EGCG analogs

**Figure 1.**
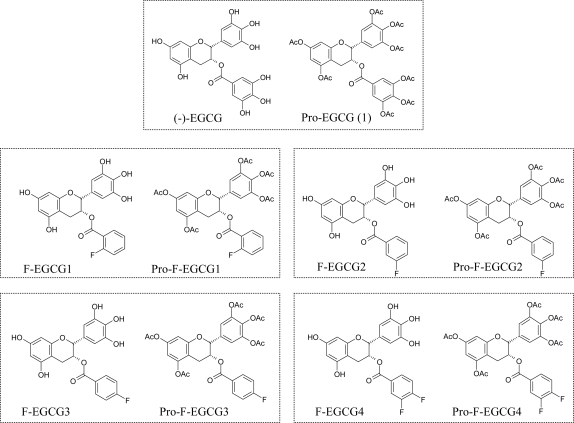
Chemical structures of EGCG and F-EGCGs as well as their pro-drugs.

In order to discover more stable and more potent tea polyphenol-based proteasome inhibitors, we synthesized several novel fluoro-substituted benzoates of EGC (F-EGCGs), which mimics the structure of EGCG (Fig. 1).

We first tested the effect of these compounds on inhibiting the chymotrypsin-like activity of a purified 20S proteasome using purified EGCG as a positive control. Each of these compounds was incubated with a purified rabbit 20S proteasome and a fluorogenic substrate for chymotrypsin-like activity for 2 h. The half-maximal inhibitory concentration or IC_50_ was then determined. (-)-EGCG showed potent proteasome-inhibitory activity with an IC_50_ of 0.68 μM. The fluoro-substituted benzoates of EGC at either meta- (F-EGCG2) or ortho-position (F-EGCG1) on the phenyl ring were potent inhibitors of purified 20S proteasomes, with IC_50_ values of 0.84 and 1.25 μM, respectively (Kui *et al*., to be submitted), similar to, or a little weaker than that of EGCG. Although the analog with the fluorophenyl group at para-position (F-EGCG3) showed a slightly decreased potency IC_50_ 1.90 μM), the difluoro-substituted benzoate of EGC at both meta- and para-positions (F-EGCG4) on the phenyl ring was a very potent inhibitor of purified 20S proteasomes IC_50_ 0.65 μM). These data demonstrate that fluoro-substituted benzoates of EGC are similar to EGCG as potent proteasome inhibitors.

### 2.2. Induction of cell death in human leukemia cells by peracetates of fluoro-substituted benzoates of EGC

Under physiological conditions, EGCG is unstable and poorly absorbed, and therefore has low bioactivity in cells [9]. Similar, the fluoro-substituted benzoates of EGC or F-EGCGs, including F-EGCG1 to F-EGCG4, showed low cell-killing activities (data not shown). We have previously shown that peracetate-protected EGCG, Pro-EGCG (1), (Fig. 1) has improved bioactivity compared with EGCG [12]. We therefore synthesized the acetate-protected form of F-EGCGs, namely Pro-F-EGCGs (Fig. 1) to elucidate whether protection of the reactive −OH groups would also improve their stability and potency.

**Figure 2.**
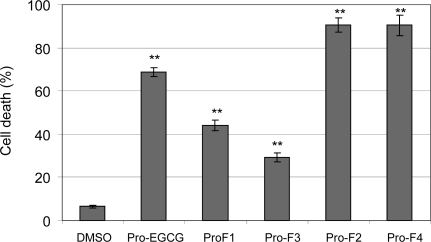
Peracetate-protected F-EGCG analogs induce cell death in human leukemia cells. Jurkat T cells were treated with 50 μM of Pro-F-EGCG1 to Pro-F-EGCG4 (indicated by Pro-F1 to Pro-F4, respectively) for 24 h, Pro-EGCG (1) and DMSO used as controls, followed by Trypan blue dye exclusion assay. The data represented are the mean number of dead cells over total cell population. ** P < 0.01, Bars, SD, mean of three experiments.

We first tested whether these Pro-F-EGCGs could induce cell death in tumor cells. Human leukemia Jurkat T cells were treated with 50 μM of each analog for 24 h, followed by Trypan-blue exclusion assay. Blue cells and cells with apoptosis-associated morphological changes (shrunken, blebbing, *etc*) were scored as dead cells. Pro-EGCG (1) and DMSO were used as controls. The results revealed that the order of potency was: Pro-F-EGCG4 (inducing 90% cell death), Pro-F-EGCG2 (90%) > Pro-F-EGCG1 (44%) > Pro-F-EGCG3 (30%) (Fig. 2). This was consistent with the order of proteasome-inhibitory potency of their unprotected compounds: F-EGCG4 IC_50_ 0.65 μM), F-EGCG2 IC_50_ 0.84 μM) > F-EGCG1 IC_50_ 1.25 μM) > F-EGCG3 IC_50_ 1.90 μM), suggesting that these prodrugs have converted into free forms of fluoro-substituted benzoates of EGCG that inhibit the cellular proteasome activity and induce cell death (see below).

In this experiment, the prodrug of EGCG, Pro-EGCG (1), induced ~70% of cell death (Fig. 2). Therefore, both Pro-F-EGCG4 and Pro-F-EGCG2 are more potent than Pro-EGCG (1). We then further studied the proteasome-inhibitory and apoptosis-inducing potencies of Pro-F-EGCG4, using Pro-EGCG (1) as a comparison.

### 2.3 Pro-F-EGCG4 inhibits tumor cellular proteasomal activity and induces apoptosis in a time-dependent manner

**Figure 3.**
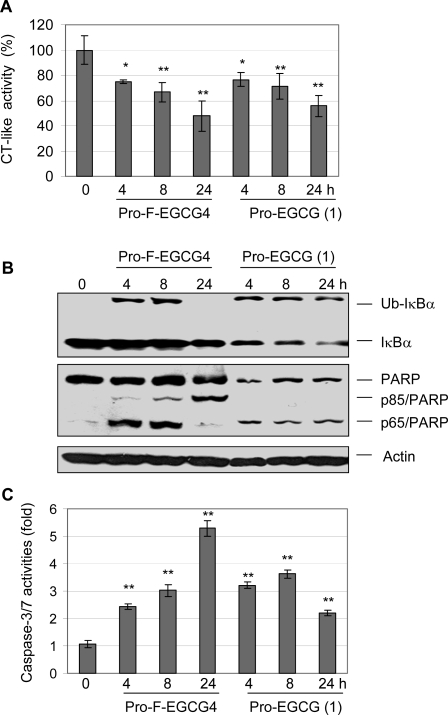
The proteasome-inhibitory and apoptosis-inducing effects of Pro-F-EGCG4 in Jurkat T cells. Jurkat T cells were treated with 50 μM of Pro-F-EGCG4 or Pro-EGCG (1) as control for indicated hours, followed by preparation of cell extracts. A, The proteasomal CT-like activity was inhibited by treatment of Pro-F-EGCG4 and Pro-EGCG (1). B, Western blot analysis showed that ubiquitinated IκB-α (p56) and cleavage of PARP (p85 and p65) in cells treated with Pro-F-EGCG4 and Pro-EGCG (1). C, Increased caspase-3/7 activation showed in Jurkat cells treated with Pro-F-EGCG4 and Pro-EGCG (1). * P < 0.05, ** P < 0.01, Bars, SD, mean of three experiments.

To study whether the induction of cell death by Pro-F-EGCG4 was due to its proteasome-inhibitory and apoptosis-inducing activities, Jurkat T cells were treated with 50 μM of Pro-F-EGCG4 and Pro-EGCG (1) as a control for 4, 8 and 24 h, followed by measurement of proteasome inhibition and apoptotic cell death. Proteasome inhibition by Pro-F-EGCG4 was confirmed by the decreased level of the proteasomal chymotrypsin (CT)-like activity (Fig. 3A). In addition, treatment of Pro-F-EGCG4 induced the appearance of the p56 IκB-α after 4 or 8 h (Fig. 3B). Previously, we reported that the p56 is an ubiquitinated form of IκB-α protein as a marker of proteasome inhibition, as verified by the immunoprecipitation-Western blot assay [13]. However, in the cells treated with Pro-F-EGCG4 for 24 h, the p56 IκB-α was not detected (Fig. 3B), suggesting the transient nature of this ubiquitinated protein accumulation. Increased level of apoptosis-specific caspase-3/7 activities was observed from 2.5 to 5.3 folds in cells treated with Pro-F-EGCG4 for 4 to 24 hours (Fig. 3C). Consistently, PARP cleavage fragment p85 was detected in Jurkat T cells treated with Pro-F-EGCG4 in a time-dependent manner (Fig. 3B). A p65 PARP fragment was also detected in cells treated with Pro-F-EGCG4 (Fig. 3B), indicating calpain activation occurred as well [14]. Apoptotic morphological changes were observed after 4 hours of treatment with Pro-F-EGCG4 (data not shown).

Treatment of Pro-EGCG (1) also caused a decrease in the level of the proteasomal CT-like activity in a time-dependent manner (Fig. 3A) and induced the increased level of the ubiquitinated form of IκB-α at all the time points (Fig. 3B). Treatment of Pro-EGCG (1) also resulted in caspase activation (increase of 2–3.5 folds) (Fig. 3C) and calpain activation, as shown by the appearance of p65 PARP fragment (Fig. 3B). We noticed that the slightly increased caspase activity was not associated production of p85 PARP (Fig. 3B), suggesting that activation of calpain is predominant (which cleaves PARP into p65 fragment, Fig. 3B) under the experimental conditions. However, under each condition, Pro-F-EGCG4 showed greater potency than Pro-EGCG (1) (Fig. 3).

### 2.4 Pro-F-EGCG4 inhibits tumor cellular proteasomal activity and induces apoptosis in a dose-dependent manner

**Figure 4.**
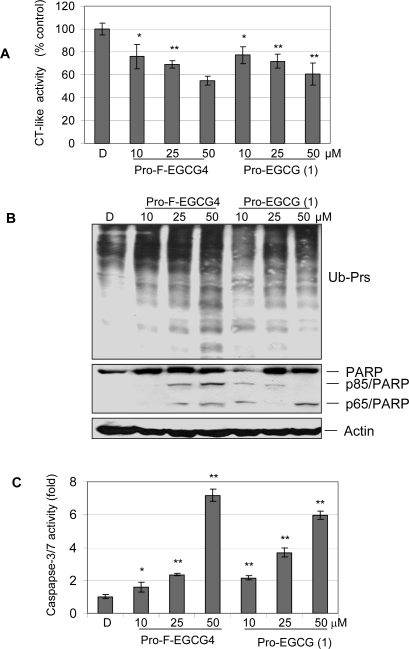
The proteasome-inhibitory and apoptosis-inducing effects of Pro-F-EGCG4 and Pro-EGCG (1) in dose-dependent manner. Jurkat T cells were treated with indicated concentrations of Pro-F-EGCG4 and Pro-EGCG (1) for 24 hours, DMSO (D) as solvent control, followed by preparation of cell extracts for measuring the chymotrypsin-like activity (*A*), Western blots analysis (*B*) and caspase-3/7 activities. * P < 0.05, ** P < 0.01, Bars, SD, mean of three experiments.

We then performed a dose-dependent experiment. Jurkat T cells were treated with Pro-F-EGCG4 or Pro-EGCG (1) at 10, 25, or 50 μM for 24 h, followed by measurement of proteasome inhibition and apoptotic cell death. Pro-F-EGCG4 inhibited the proteasomal CT-like activity in a dose-dependent manner (Fig. 4A). Consistently, Pro-F-EGCG4 caused accumulation of ubiquitinated proteins in a dose-dependent manner (Fig. 4B). Furthermore, caspase activation and PARP cleavage were observed in Jurkat T cells treated with Pro-F-EGCG4 in a dose-dependent manner (Fig. 4B–C).

Pro-EGCG (1) also showed inhibition of the proteasomal CT-like activity, accumulation of ubiquitinated proteins, and activation of caspase activity (Fig. 4). In this dose-dependent experiment, Pro-EGCG (1) at 50 μM was able to induce caspase 3/7 activity by 6-fold, which was not seen in the kinetic experiment under similar conditions (Fig. 3). This discrepancy is due to the fact that when very apoptotic cells were used for protein extract preparation, loss of proteins (including caspases) would give a lower activity measurement under *in vitro* conditions. The conditions of cell culture, the drug stability, and other factors in each experiment will affect the profile of *in vitro* caspase activity. Regardless of that, Pro-EGCG (1) was less potent than Pro-F-EGCG4 in the dose-dependent experiment (Fig. 4). Therefore, these results demonstrated that synthetic protected fluoro-substituted benzoates of EGC achieved improvement of their biological activities over natural EGCG and Pro-EGCG (1) [12].

The ubiquitin/proteasome-dependent degradation pathway plays an essential role in up-regulation of cell proliferation, down-regulation of cell death, and development of drug resistance in human tumor cells. Therefore, proteasome inhibitors show great potential as novel anticancer drugs [2–3].

Previously, we have reported that EGCG is a natural inhibitor of proteasomal chymotrypsin-like activity [4–5]. However, EGCG, especially on its hydroxyl groups, is subject to extensive biotransformation including methylation by catechol-O-methyltransferase (COMT) [15] and glucuronidation by UDP-glucuronosyltransferase (UGT) [16]. It has been reported that the biotransformation reduces the bioavailability and stability of EGCG. We found that methylation of EGCG could decrease its proteasome-inhibitory activity, contributing to decreased cancer-preventive effects of tea consumption under physiological conditions [17].

In order to improve the bioavailability of EGCG, we synthesized peracetate-protected (-)-EGCG molecule, Pro-EGCG (1). *In vitro* results showed that the cellular permeability and stability of Pro-EGCG (1) significantly enhanced, compared with natural EGCG. It was further confirmed by HPLC analysis that Pro-EGCG (1) could convert to (-)-EGCG in cultured human leukemic Jurkat T cells [17]. The data from animal study also demonstrated that Pro-EGCG (1) possessed greater anti-tumor activity in human breast cancer xenografts in mice, compared with natural EGCG [12].

Encouraged by the *in vitro* and *in vivo* results of Pro-EGCG (1), we synthesized more EGCG analogues and tried to discover the analogs with greater potency in the proteasome inhibition and apoptosis induction in cancer cells. We modified the D-ring of EGCG by adding one or two fluorine(s), and also made their acetate-protected prodrugs.

By testing this series of EGCG analogs, we found that Pro-F-EGCG4 was most potent. The results from current studies show that Pro-F-EGCG4 has improved potency compared to Pro-EGCG (1) to inhibit the proteasomal chymotrypsin-like activity, leading to accumulation of proteasome target proteins (such as IκB-α) and apoptosis in human leukemia Jurkat T cells, as measured by activation of caspases and PARP cleavage. This result indicates that Pro-F-EGCG4 may be more stable or should have better bioavailability than EGCG and Pro-EGCG (1). These data suggest that the peracetate-protected fluoro-substituted benzoates of epigallocatechin have the great potential to be developed into novel anti-cancer and cancer-preventive agents. We will further confirm bioeffects of Pro-F-EGCG4 in animal models.

## 3. Experimental Section

### 3.1. Materials

Highly purified (-)-EGCG and dimethyl sulfoxide (DMSO) were purchased from Sigma-Aldrich. Purified 20S proteasome (rabbit) was purchased from Boston Biochem. Fluorogenic peptide substrate Suc-Leu-Leu-Val-Tyr-AMC (for the proteasomal chymotrypsin-like activity) was obtained from Calbiochem. Polyclonal antibody to ubiquitin, IκB-α̣ monoclonal anti-actin, anti-goat, anti-rabbit, and anti-mouse IgG-horseradish peroxidase were obtained from Santa Cruz Biotechnology Inc. Mouse monoclonal anitbody against human poly(ADP-ribose) polymerase (PARP) was purchased from Biomol International LP (Plymouth Meeting, PA). RPMI 1640, Penicillin and streptomycin were purchased from Invitrogen.

### 3.2. Synthesis of F-EGCG analogs and their prodrugs

We have designed and semi-synthesized four fluoro-substituted benzoates of EGC as EGCG analogs, named F-EGCG1, F-EGCG2, F-EGCG3 and F-EGCG4, respectively (Fig. 1). The synthesis and biological evaluation of these four F-EGCG analogs will be published elsewhere (Sun Dong Kui *et al*., to be submitted). The peracetates or prodrugs of F-EGCG1, F-EGCG2, F-EGCG3 and F-EGCG4 (named as Pro-F-EGCGs) were subsequently synthesized according to the following procedure.

**(-)-(*2R, 3R*)-5,7-Diacetoxy-2-(3,4,5-triacetoxyphenyl)chroman-3-yl 2-fluorobenzoate (Pro-F-EGCG1)**. Under an N_2_ atmosphere, to a solution of (-)-(*2R, 3R*)-5,7-dihydroxy-2-(3,4,5-trihydroxyphenyl)chroman-3-yl 2-fluorobenzoate (20 mg, 0.047 mmol) in pyridine (1 ml), acetic anhydride (0.2 ml) was added dropwise at 0°C. The reaction mixture was stirred at room temperature overnight. The excess pyridine was distilled under vacuum. The residue was purified by flash chromatograph on silica gel (EtOAc/n-hexane, 1/1 in v/v) to afford (-)-(*2R, 3R*)-5,7-Diacetoxy-2- (3,4,5-triacetoxyphenyl)chroman-3-yl 2-fluorobenzoate (28 mg, 94.0% yield). mp: 89–90ºC; [α]_D_=−52.1 (c=1.0, CHCl_3_); ^1^H NMR (CDCl_3_, 600 MHz) δ 7.67 (m, 1 H), 7.45 (m, 1 H), 7.31 (bs, 2 H), 7.12 (m, 1 H), 7.05 (m, 1 H), 6.73 (d, J=2.34 Hz, 1 H), 6.58 (d, J=2.34 Hz, 1 H), 5.63 (m, 1 H), 5.21 (bs, 1 H), 3.11 (dd, J=17.88, 2.28 Hz, 1 H), 3.06 (dd, J=17.88, 4.56 Hz, 1 H), 2.29–2.25 (m, 15 H). ^13^C NMR (CDCl_3_, 600 MHz) δ 168.9, 168.5, 167.6, 166.8, 163.4, 162.8, 161.1, 154.7, 149.7, 143.4, 135.5, 134.8, 134.7, 134.3, 132.2, 123.7, 117.9, 116.9, 109.5, 108.9, 107.9, 76.4, 67.8, 25.9, 21.1, 20.9, 20.6, 20.1. HRMS (ESI): calculated for C_32_H_28_FO_13_ (M+H) 639.1514, found 639.1508.

**(-)-(2R, 3R)-5,7-Diacetoxy-2-(3,4,5-triacetoxyphenyl)chroman-3-yl 3-fluorobenzoate (Pro-F-EGCG2).** Following the procedure for the preparation of Pro-F-EGCG1, the prodrug F-EGCG2 was obtained (90.6% yield). mp: 85–86ºC; [α]D=−48.6 (c=1.0, CHCl3); 1H NMR (CDCl3, 600 MHz) δ 7.68 (m, J=7.8 Hz, 2 H), 7.46 (m, 1 H), 7.31 (m, 1 H), 7.11 (bs, 2 H), 7.03(m, 1 H), 6.73 (d, J=2.28 Hz, 1 H), 6.58 (d, J=2.28 Hz, 1 H), 5.63 (m, 1 H), 5.19 (bs, 1 H), 3.09 (d, J=4.02 Hz, 1 H), 2.29–2.23 (m, 15 H) ^13^C NMR (CDCl3, 600 MHz) δ 168.9, 168.5, 167.6, 166.8, 163.4, 162.8, 161.1, 154.7, 149.7, 149.6, 143.3, 135.5, 134.3, 132.2, 124.0, 118.8, 117.9, 116.7, 109.5, 108.8, 107.9, 76.3, 67.8, 25.9, 21.0, 20.7, 20.5, 20.1. HRMS (ESI): calculated for C32H28FO13 (M+H) 639.1514, found 639.1525.

**(-)-(2R, 3R)-5,7-Diacetoxy-2-(3,4,5-triacetoxyphenyl)chroman-3-yl 4-fluorobenzoate (Pro-F-EGCG3).** Following the procedure for the preparation of Pro-F-EGCG1, the prodrug F-EGCG3 was obtained. (94.0% yield): mp: 97–99ºC; [α]D=−56.4 (c=1.0, CHCl3); 1H NMR (CDCl3, 600 MHz) δ 7.87 (m, 4 H), 7.27 (m, 1 H),7.13 (bs, 2 H),7.03 (m, 2 H), 6.74 (d, J=2.28 Hz, 1 H), 6.72 (d, J=2.28 Hz, 1 H), 5.87 (m, 1 H), 5.12 (bs, 1 H), 3.05 (m, 2 H), 2.28–2.24 (m, 15 H) ^13^C NMR (CDCl3, 600 MHz) δ 169.3, 169.1, 168.7, 168.2, 168.0, 166.7, 165.0, 155.0, 149.7, 143.4, 143.0, 135.7, 132.5, 132.4, 130.7, 125.6, 118.1, 115.6, 115.4, 112.8, 108.8, 108.0, 76.5, 67.6, 26.0, 21.1, 20.8, 20.7, 20.5, 20.3. HRMS (ESI): calculated for C32H28FO13 (M+H) 639.1514, found 639.1501.

**(-)-(2R,3R)-5,7-Diacetoxy-2-(3,4,5-triacetoxyphenyl)chroman-3-yl 3,4-difluorobenzoate (Pro-F-EGCG4).** Following the procedure for the preparation of Pro-F-EGCG1, the prodrug F-EGCG4 was obtained (91.8% yield): mp: 94–95ºC; [α]D=−58.1 (c=1.0, CHCl3); 1H NMR (CDCl3, 600 MHz) δ 7.67 (m, 1 H), 7.45 (m, 1 H), 7.28 (bs, 2 H), 7.14 (m, 1 H), 6.73 (d, J=2.28 Hz, 1 H), 6.58 (d, J=2.28 Hz, 1 H), 5.59 (m, 1 H), 5.30 (bs, 1 H), 3.07 (m, 2 H), 2.31–2.23 (m, 15 H) ^13^C NMR (CDCl3, 600 MHz) δ 168.9, 168.5, 167.6, 166.8, 163.4, 162.8, 161.1, 154.7, 149.9, 149.7, 149.6, 143.3, 135.5, 134.3, 119.2, 118.6, 116.7, 109.5, 108.8, 76.3, 67.8, 25.9, 21.0, 20.7, 20.5, 20.1. HRMS (ESI): calculated for C32H27F2O13 (M+H) 657.1419, found 657.1407.

### 3.3. Cell viability assay

The Trypan blue dye exclusion assay was performed by mixing 20 μL of cell suspension with 20 μL of 0.4% Trypan blue dye before injecting into a hemocytometer and counting [17, 18]. The number of cells that absorbed the dye and those that exclude the dye were counted, from which percentage of nonviable cell number over the total cell number was calculated.

### 3.4. Cell culture, drug treatment and protein extraction

Human leukemia Jurkat T cells were cultured in RPMI 1640 medium supplemented with 10% fetal calf serum, 100 units/mL of penicillin, and 100 μg/mL of streptomycin. Cells were maintained at 37ºC in a humidified incubator with an atmosphere of 5% CO2. Jurkat T cells were treated by selected compounds at various concentrations for indicated hours (see figure legends for details), and a whole-cell extract was then prepared as described previously [18]. Briefly, cells were harvested, washed with PBS, and homogenized in a lysis buffer (50 mM, Tris-HCl, pH 8.0, 150 mM NaCl, 0.5% NP-40, 0.5 mM penylmethylsulfonyl fluoride, and 0.5 mM dithiothreitol) at 4ºC. Afterwards, the lysates were centrifuged at 12,000 × g for 15 min at 4ºC and the supernatants collected as whole-cell extracts.

### 3.5. Inhibition of cellular proteasome activity by Pro-F-EGCG analogs

Human leukemia Jurkat T cells were treated with each compound at indicated concentration for indicated hours (see the figure legends for details), harvested, and lysed as described previously [5, 12, 18]. Whole cell extracts were prepared as detailed in section *3.4*, aliquoted and kept at −80ºC. One of the aliquots was used for determination of protein concentration. After that, whole cell extracts (10 μg) from each preparation were incubated with Suc-Leu-Leu-Val-Tyr-AMC (20 μM) fluorogenic substrate at 37°C in 100 μl of assay buffer (50 mM Tris-HCL, pH 7.5) for 2 h. After incubation, production of hydrolyzed 7-amino-4-methylcoumarin (AMC) groups was measured using a Victor^3^ Multilabel Counter with an excitation filter of 380 nm and an emission filter of 460 nm (PerkinElmer, Boston, MA, USA).

### 3.6. Induction of caspase-3 activity by Pro-F-EGCG analogs

Cells were treated with each compound at indicated concentration for indicated hours, harvested, and lysed as described previously [5]. Ac-DEVD-AMC (40 μM) was then incubated with the prepared cell lysates for 2 h and the caspase-3 activity was measured as described previously [18].

### 3.7. Western blotting

Analysis of IκB-α, PARP, and ubiquitinated proteins was performed using monoclonal or polyclonal antibodies, according to previously reported protocols [5].
